# Elucidating the pharmacological foundations and mechanisms of the Sihai Shuyu formula in treating Graves’ disease through integrated serum metabolomics and network pharmacology with molecular docking techniques

**DOI:** 10.3389/fendo.2025.1511808

**Published:** 2025-01-30

**Authors:** Xiaoju Liu, Xingjia Li, Wenbin Huang, Yifan Cui, Fengyun Cheng, Guofang Chen, Xiaodong Mao, Chao Liu, Shuhang Xu

**Affiliations:** ^1^ Endocrine and Diabetes Center, Affiliated Hospital of Integrated Traditional Chinese and Western Medicine, Nanjing University of Chinese Medicine, Jiangsu Province Academy of Traditional Chinese Medicine, Nanjing, China; ^2^ Key Laboratory of TCM Syndrome and Treatment of Yingbing (Thyroid Disease) of State Administration of Traditional Chinese Medicine, Jiangsu Province Academy of Traditional Chinese Medicine, Nanjing, China

**Keywords:** Graves’ disease, Sihai Shuyu Formula, network pharmacology, molecular docking, IGF1R/PI3K/Akt signaling pathway

## Abstract

**Ethnopharmacological relevance:**

The Sihai Shuyu Formula (SHSY) shows promising potential for treating Graves’ disease (GD), although the therapeutic mechanisms and pharmacological basis of SHSY have not been thoroughly evaluated.

**Objective:**

This work is aim to investigate the pharmacological basis and mechanism of SHSY in the treatment of GD by integrating non-targeted serum metabolomics and network pharmacology coupled with molecular docking technology.

**Materials and methods:**

GD was induced in mice through injections of Ad-TSH289. Treatments included methimazole, inorganic iodine, and both low and high doses of SHSY administered via gavage. At the end of the treatment period, serum levels of thyroxine (T4) and thyrotropin receptor antibody (TRAb) were measured. Hematoxylin-Eosin (H&E) staining assessed the effects of these pharmacological interventions on thyroid gland tissues. Ultra-High Performance Liquid Chromatography with Quadrupole Time-of-Flight Mass Spectrometry (UPLC-Q-TOF-MS) was used in conjunction with network pharmacology and molecular docking to identify and predict SHSY’s active chemical components and targets. A comprehensive analysis of the multi-level bioinformatic analysis, including protein-protein interactions (PPI) and functional pathways of the targets, was conducted, followed by verification through immunohistochemistry (IHC) to clarify SHSY’s pharmacological basis and action mechanisms in treating GD.

**Results:**

After 8 weeks of treatment, SHSY significantly reduced serum T4 and TRAb levels in GD mice and enhanced the morphology of thyroid tissues. Comparative analysis of rat blood samples and SHSY using UPLC-Q-TOF-MS identified 19 blood-entry components, the potential active components of SHSY acting on GD. Further network pharmacological analysis indicated that SHSY targets the PI3K/Akt signaling pathway through components such as PIK3CD, SRC, PIK3CA, HRAS, EGFR, PIK3R1, AKT1, PTPN11, and PIK3CB. Molecular docking confirmed the effective binding of SHSY’s components to these targets. IHC confirmed that the IGF1R/PI3K/Akt signaling pathway is a significant therapeutic target of SHSY, with key substances including Guggulsterone, Betulinic aldehyde, and Forsythoside H.

**Conclusions:**

SHSY appears to effectively treat GD through the IGF1R/PI3K/Akt signaling pathway, with Guggulsterone, Betulinic aldehyde, and Forsythoside H as the critical pharmacological components. It may serve as an adjunctive treatment for GD alongside traditional therapies such as antithyroid medications, surgery, and radioiodine therapy.

## Introduction

Hyperthyroidism, a thyrotoxic condition caused by improper synthesis and secretion of thyroid hormones, is most commonly attributed to Graves’ disease (GD) (1). Untreated, GD can lead to severe complications such as osteoporosis, atrial fibrillation, and even heart failure in rare instances (2–4). The current primary treatments for GD are anti-thyroid drugs (ATDs), radioiodine therapy (RIT), and surgery. ATDs, the first-line treatment, requires a lengthy course of 18-24 months with frequent monitoring of thyroid function. However, it can cause adverse effects such as leukopenia, impaired liver function, and vasculitis (5). RIT, effective at achieving euthyroidism, can worsen thyroid eye disease and may elevate cancer risk (6, 7). Thyroidectomy necessitates lifelong thyroid hormone replacement and poses risks of recurrent laryngeal nerve injury, hematoma, and hypoparathyroidism (8). Thus, there is a continuing need to explore new treatment modalities for GD.

Historically, hyperthyroidism was categorized among the “galls” in ancient Chinese medicine. The causes of gall disease are related to a variety of factors, including innate endowment, emotional and emotional internal injuries are the most closely (9), and Qi stagnation, phlegm congealment and blood stasis are the main disease pathologies. Therefore, several scholars believe that the treatment of gall disease should start from the liver (10). Formulations for gall diseases often included iodine-rich traditional Chinese medicines like sargassum and thalluslaminariae, achieving significant results (11). Recent studies confirm that combining iodine-rich herbs with ATDs is both effective and safe for managing hyperthyroidism (12). The 2021 expert consensus on iodine-rich traditional Chinese medicine for treating GD underscores its clinical superiority (13).

Sihai Shuyu Pill, a renowned Qing Dynasty prescription for gallbladder treatment (14), which has the efficacy of eliminating galls and dispersing knots, promoting circulation of Qi and dispersing the liver. It has been demonstrated to induce apoptosis in the thyroid tissue of hyperthyroid rats, thus mitigating hyperthyroidism symptoms (15). The SHSY, derived from the Sihai Shuyu Pill, comprises sargassum (Hai zao), thalluslaminariae (Kun bu), endoconcha sepiae (Hai piao xiao), goncha meretricis seu cyclinae (Ge ke), citrus reticulata (Chen Pi), and aucklandiae radix (Mu Xiang). In this study, we assessed SHSY’s efficacy in adenovirus-induced GD mice by measuring serum T4 and TRAb levels. Utilizing ultra-high performance liquid chromatography with quadrupole time-of-flight mass spectrometry (UPLC-Q-TOF-MS), network pharmacology, and molecular docking techniques, we identified, screened, and predicted the active chemical components and targets of SHSY. We analyzed its PPI, target functional pathways, and other multi-level bioinformatic analysis, and then verified it by IHC, to elucidate the pharmacological basis and mechanism of the SHSY in treating GD.

## Materials and methods

### Drug preparation

SHSY preparation: The formulation consists of Sargassum (15 g), Thallus laminariae (15 g), Endoconcha sepiae (6 g), Goncha meretricis seu cyclinae (3 g), Citrus reticulata (9 g), and Aucklandiae radix (6 g). The ingredients must conform to the standards specified in the 2020 version of the “Chinese Pharmacopoeia”, which includes guidelines for the detection of medicinal herbs and tablets. All herbs were sourced from the Affiliated Hospital of Integrated Traditional Chinese and Western Medicine, Nanjing University of Chinese Medicine. Each ingredient was placed into 5-liter round-bottomed flasks, 10 and 8 times their weight in water were added to decoct twice, each for 1.5 hours. The decoctions were combined, concentrated to a density of 1.08 g/ml, and stored at 4°C.

### Animals and interventions

A total of 120 six-week-old female BALB/c mice, purchased from the Center for Comparative Medicine at Yangzhou University [License No. SYXK (SU) 2022-0009], were housed in the Laboratory Animal Center of the Affiliated Hospital of Integrated Traditional Chinese and Western Medicine, Nanjing University of Chinese Medicine [License No. SYXK: (SU) 2021-0025] under conditions of 25 ± 2°C and a 12-hour light/dark cycle. The mice had ad libitum access to food and water. All procedures involving animals were conducted in compliance with the guidelines of the Animal Ethics Committee of the Affiliated Hospital of Integrated Traditional Chinese and Western Medicine, Nanjing University of Chinese Medicine (AEWC-20230603-314).

The mice were randomly divided into 7 groups: control (Con) group, adenovirus expressing green fluorescent protein (Ad-GFP) group, model (Mod) group, methimazole (MMI) group, inorganic iodine (KI) group, SHSY low-dose (SHSY-L) group, and SHSY high-dose (SHSY-H) group. The Ad-GFP group received 10^10^ units of Ad-GFP, while the Con group received an equivalent volume of saline. The remaining mice received four intramuscular injections of adenovirus expressing the human TSH receptor at three-week intervals, with 10^10^ units per injection. Thirteen weeks after the first injection, orbital venous blood was collected to measure T4 and TRAb levels and assess the modeling rate (16). Mice in the MMI group were orally administered 3 mg/kg/day of MMI (Merck, Germany); those in the SHSY-L group received 11 g/kg/day of SHSY via gavage, and the SHSY-H group received 22 g/kg/day of SHSY. Blood and thyroid samples were collected after anaesthesia by intraperitoneal injection using 1% sodium pentobarbital at a dose of 50 mg/kg.

Six SPF-grade male SD rats, each weighing 200 ± 10 g, were purchased from Spivey (Beijing) Biotechnology Co. Ltd [License No. SCXK (Beijing) 2019-0010] and housed in the Laboratory Animal Center of the Affiliated Hospital of Integrated Traditional Chinese and Western Medicine, Nanjing University of Chinese Medicine [License No. SYXK: (Su) 2021-0025]. The study protocol and procedures received approval from the Animal Ethics Committee of the Affiliated Hospital of Integrated Traditional Chinese and Western Medicine, Nanjing University of Chinese Medicine (AEWC-20230603-314).

Following seven days of acclimation, six SD male rats were randomly assigned to two groups based on body mass: a Con group (3 rats) and an SHSY group (3 rats), both with unrestricted access to food and water. After three days of treatment, the rats were fasted for 12 hours without water prior to blood sampling. Orbital venous blood was collected at 0.5, 1, 2, 3, and 4 hours post-gavage. The samples were collected in anticoagulated EP tubes and centrifuged at 3000 rpm for 10 minutes within 30 minutes of collection to obtain plasma, which was then pooled on a per-rat basis.

### Enzyme-linked immunosorbent assay

Samples and controls were allocated to each well for assay. Samples were initially incubated at 37°C for 90 minutes, followed by the addition of the prepared biotinylated antibody working solution and a second incubation at 37°C for 60 minutes. Subsequently, the enzyme-conjugated working solution was added and the samples were incubated at 37°C in the dark for 30 minutes. TMB color development working solution was then added under dark conditions at 37°C for 20 minutes to ensure accurate colorimetric development. The optical density (OD) of each sample was immediately measured using an enzyme marker after adding a stop solution. A standard curve was plotted, and levels of serum T4 and TRAb were quantified.

### H&E staining and IHC

Thyroid tissue was fixed in paraformaldehyde, dehydrated, and embedded in paraffin. Sections approximately 4μm thick were treated with xylene and ethanol and stained using an H&E kit.

Paraffin-embedded thyroid tissue sections from each group were cut to 4 μm for IHC staining. After pretreatment with xylene, sections were incubated overnight at 4°C with primary antibodies against IGF1-R (Proteintech, 20254-1-AP), PI3K (Proteintech, 67071-1-lg), and AKT (Proteintech, 60203-2-lg). Following this, sections were incubated with complement, HRP-conjugate, and DAB, and images were captured using an Olympus microscope.

### Instruments and reagents

Instruments: Ultrasonic Cleaner (F-060SD, Shenzhen Fuyang Technology Group Co., Ltd.), Vortex Oscillator (TYXH-I, Shanghai Khannuo Instrument Co., Ltd.), Benchtop High-Speed Freezing Centrifuge (TGL-16MS, Shanghai Lu Xiangyi Centrifuge Instrument Co., Ltd.), High Performance Liquid Chromatograph (ACQUITY UPLC I-Class HF, Waters), Chromatography Columns (ACQUITY UPLC HSS T3 (100 mm×2.1 mm, 1.8 um), Waters), PDA detector (ACQUity UPLC, Waters), high-resolution liquid-mass spectrometer (Thermo-Obritrap-QE, Thermo).

Reagents: Methanol (A452-4, Thermo Fisher Scientific), acetonitrile (A998-4, Thermo Fisher Scientific), formic acid (A117-50, Thermo Fisher Scientific), and pure water.

### Chromatographic conditions

The chromatographic column used was the ACQUITY UPLC HSS T3 (100 mm × 2.1 mm, 1.8 μm); mobile phase consisted of A-water (containing 0.1% formic acid) and B-acetonitrile; gradient elution schedule: 0-2 min, 5% B; 2-4 min, 5%-30% B; 4-8 min, 30%-50% B; 8-10 min, 50%-80% B; 10 ~15 min, 80%-100% B; 15-16 min, return to 5% B. The column temperature was 45°C; flow rate: 0.35 mL/min; injection volume: 5 μL; PDA scan range: 210-400 nm.

### Mass spectrometry conditions

Sample mass spectrometry signal acquisition was conducted in both positive and negative ion scanning modes using a HESI source. Detection modes included positive and negative ion settings; sheath gas flow rate: 35Arb; auxiliary gas flow rate: 8Arb; spray voltage was set at 3800 V for positive ion mode and 3000 V for negative ion mode; capillary temperature: 320°C; S-lens RF level: 50; auxiliary gas heater temperature: 350°C. Data acquisition mode: Full MS/dd-MS2 Top 8; MS scanning range: m/z 100 to 1200; full MS scanning resolution: 70,000, MS/MS scanning resolution: 17,500; collision energies were 10 eV, 20 eV, and 40 eV.

### Network pharmacology and molecular docking

Using the Swiss Target Prediction database, potential targets of the blood-entry components of SHSY were predicted with a probability value > 0 as the screening criterion. The OMIM, Disgenet, Drugbank, and GeneCards databases were searched using the keyword “Graves’ disease”. Data from these four databases were integrated and filtered to identify disease-related targets for GD. To determine the key targets for GD treatment by SHSY, a Veen diagram was created using Bioinformatics tools. The identified targets were then uploaded to the STRING database, setting the species to “Homo sapiens” and the interaction confidence threshold to the highest confidence level (0.7). This process generated a map of protein interactions. These results were imported into Cytoscape 3.7.2 to construct PPI network diagrams. The DAVID database was utilized for Gene Ontology (GO) and Kyoto Encyclopedia of Genes and Genomes (KEGG) enrichment analysis, with the findings presented visually. Component attributes and drug-disease common targets were then identified and visualized in Cytoscape 3.7.2. Lastly, using the Pubchem database, Alpha-Fold database, and ChemDraw software, CB-dock2 was employed to illustrate the binding energy and molecular docking of significant active ingredients and core targets in drug-disease treatment, if the affinity value is <-5.0 kCal/mol, it indicates that the two have good binding ability (17, 18).

### Data analysis

Data were analyzed using SPSS 25.0, expressed as mean ± SD. Multiple comparisons were conducted using one-way ANOVA followed by Bonferroni’s *post hoc* test and statistical significance set at *P*<0.05/(9 comparisons) = 0.0056.

## Results

### SHSY effectively alleviates the condition of GD mice

Thirteen weeks after the initial administration of adenovirus, serum T4 and TRAb levels were measured to assess the modelling status of GD mice. Mice demonstrating successful modeling were randomly divided into Mod, MMI, KI, SHSY-L, and SHSY-H groups. After an eight-week intervention, thyroid tissue and blood samples from the mice were collected for H&E staining and measurement of serum T4 and TRAb levels.

**Figure 1:**
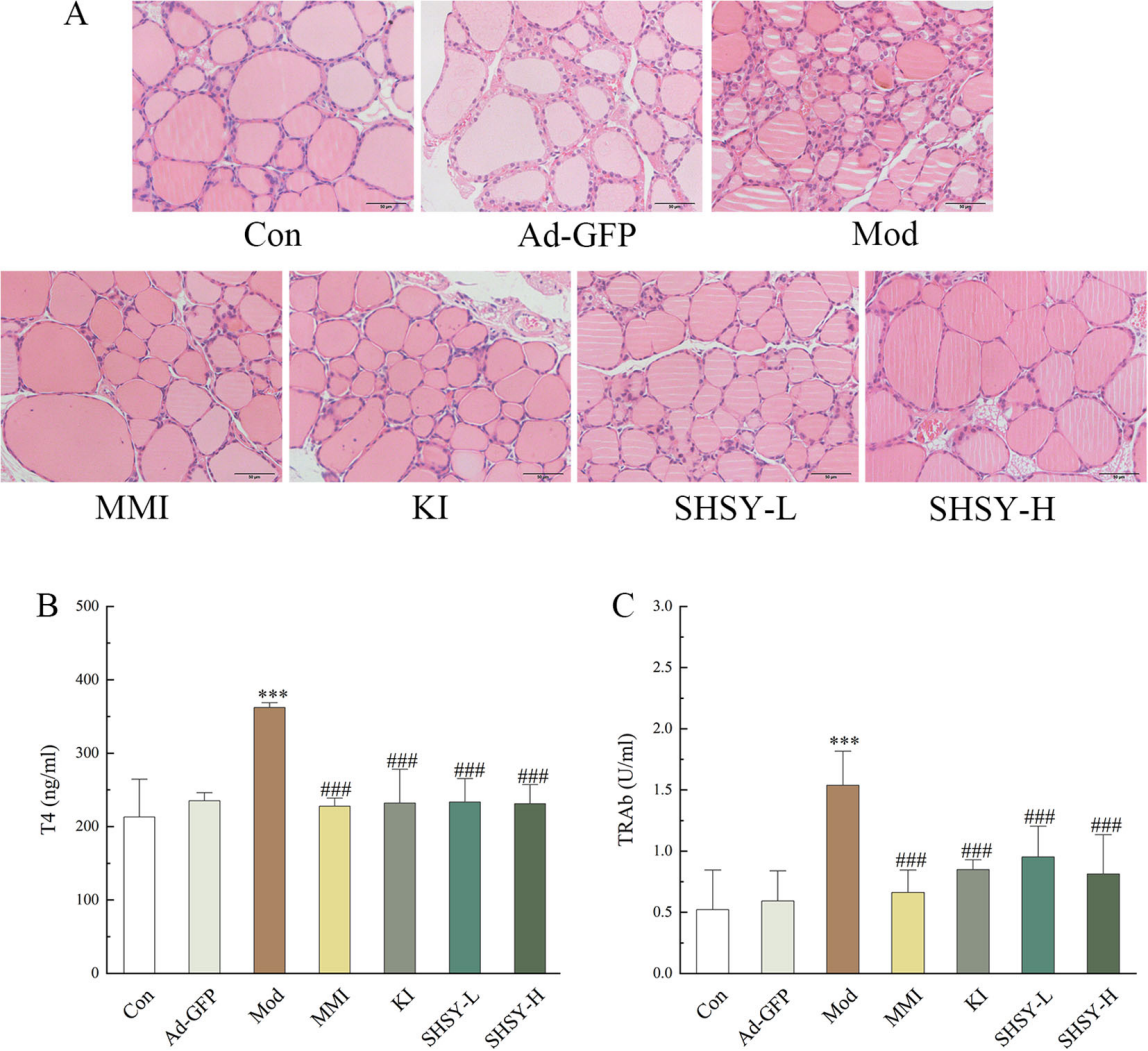
SHSY relieve GD in mice. In comparison to the Con group, the thyroid follicular cells in the Mod group display disordered arrangements and irregular shapes, which are ameliorated by the administration of MMI, KI, SHSY-L, and SHSY-H **(A)**. Serum T4 and TRAb levels are higher in the Mod group than in the Con group and decreased following treatment **(B, C)**. Compared to the Con group, ***P<0.001; compared to the Mod group, ^###^ *P*<0.001.

H&E staining showed significant morphological changes in the thyroid follicular epithelial cells of mice in the Mod group, which were highly columnar or cuboidal with varying sizes, disordered arrangement, and irregular shapes. In contrast, thyroid follicular epithelial cells from GD mice treated with MMI, KI, SHSY-L, and SHSY-H exhibited a flattened morphology with similar sizes and neatly arrangement (**Figure 1A**). Serological analysis indicated that T4 levels were significantly elevated in the Mod group (362.03 ± 8.13 ng/ml) compared to the Con group (213.02 ± 49.12 ng/ml, *P*<0.001). Treatment with MMI, KI, SHSY-L, and SHSY-H significantly reduced T4 levels to 227.75 ± 12.22 ng/ml, 231.87 ± 38.19 ng/ml, 233.27 ± 20.22 ng/ml, and 231.14 ± 28.82 ng/ml, respectively, all *P*<0.001. No significant differences were observed between the SHSY-L and SHSY-H groups, and no significant differences between the SHSY and MMI, KI group (**Figure 1B**). Similarly, TRAb levels were significantly higher in the Mod group (1.54 ± 0.18 U/ml) compared to the Con group (0.52 ± 0.19 U/ml, *P*<0.001), and significantly decreased to 0.66 ± 0.23 U/ml, 0.85 ± 0.06 U/ml, 0.95 ± 0.24 U/ml, and 0.81 ± 0.26 U/ml respectively after treatment with MMI, KI, SHSY-L, and SHSY-H, all *P*<0.001, with no significant differences observed between the SHSY-L and SHSY-H groups, and no significant differences between the SHSY and MMI, KI group (**Figure 1C**). This suggests that SHSY effectively reversed the histopathological changes in the thyroid glands of GD mice and significantly reduced T4 and TRAb levels, thereby alleviating.

### Identification of blood-entry components of SHSY

**Figure 2:**
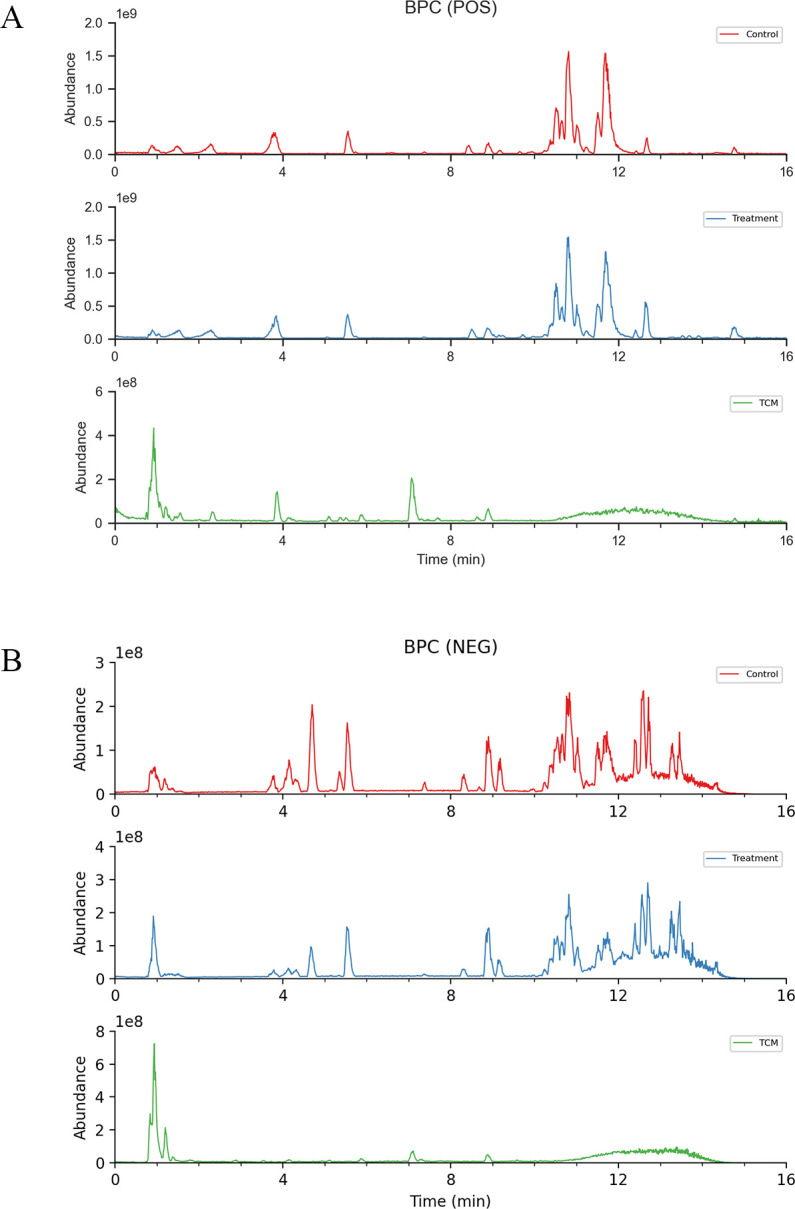
Total ion chromatogram of constituents in GD mice serum after oral administration of SHSY. **(A)** Positive ion mode. **(B)** Negative ion mode.

Utilizing the UHPLC-ESI-QE-Orbitrap-MS method (**Figure 2**), 19 blood-entry components of SHSY were preliminarily identified by comparing chromatographic retention times, mass spectrometry cleavage patterns, precise molecular weights, and fragmentation ion data with those of controls or components reported in the literature. These components include Atractylenolide II, Forsythoside H, Guggulsterone, Betulinaldehyde, etc. (**Table 1**).

**Table 1:** Analysis of constituents in GD mice serum following oral administration of SHSY.

No.	Rt/min	Metabolites	Formula	Ion mode	Theoretical mass	Measured mass	Error/ppm
1	10.7	Atractylenolide II	C_15_H_20_O_2_	POS	233.153606	233.1537	0.29
2	5.56	Elemicin	C_11_H_14_O_6_S	NEG	273.0438326	273.0438	-0.14
3	4.72	Forsythoside H	C_29_H_36_O_15_	NEG	623.1981451	623.1988	1.04
4	10.68	Incensole Acetic acid	C_22_H_34_O_5_	NEG	359.2227883	359.2229	0.27
5	6.03	Isocurcumenol	C_21_H_28_O_9_	POS	447.1625654	447.1623	-0.53
6	9.05	Marrubiin	C_20_H_28_O_6_	NEG	409.1867547	409.1871	0.82
7	5.82	Myristicin	C_10_H_12_O_6_S	NEG	259.0281828	259.0282	0.1
8	11.3	N-Benzyllinoleamide	C_25_H_39_NO_2_	POS	408.2873987	408.2856	-4.29
9	7.16	Rupestonic acid	C_15_H_20_O_3_	NEG	247.1339674	247.1338	-0.69
10	8.24	Guggulsterone	C_21_H_32_O_5_S	NEG	395.1897686	395.1897	-0.11
11	6.82	Farnesol	C_21_H_32_O_9_	POS	451.1938548	451.1938	-0.07
12	11.41	beta-Mangostin	C_25_H_28_O_7_	NEG	439.1762289	439.1771	2.06
13	6.1	Demethylsuberosin	C_20_H_24_O_10_	NEG	423.1296716	423.1301	1.11
14	4.46	2-Hydroxy-4-methoxybenzoic acid	C_7_H_6_O_4_	NEG	307.0459443	307.047	3.35
15	10.99	5-hydroxyicosa-6,8,11-trienoic acid	C_20_H_34_O_3_	POS	340.284628	340.2845	-0.42
16	8.32	Acitretin	C_26_H_32_O_9_	NEG	469.1868281	469.1877	1.93
17	5.46	Beta-Zearalanol	C_24_H_32_O_12_	NEG	493.1714626	493.1696	-3.83
18	9.78	Betulinaldehyde	C_30_H_46_O_3_	POS	455.3519721	455.3518	-0.3
19	8.45	Curzerene	C_21_H_28_O_8_	NEG	407.1711421	407.1714	0.67

### Network pharmacological analysis of SHSY for the treatment of GD

**Figure 3:**
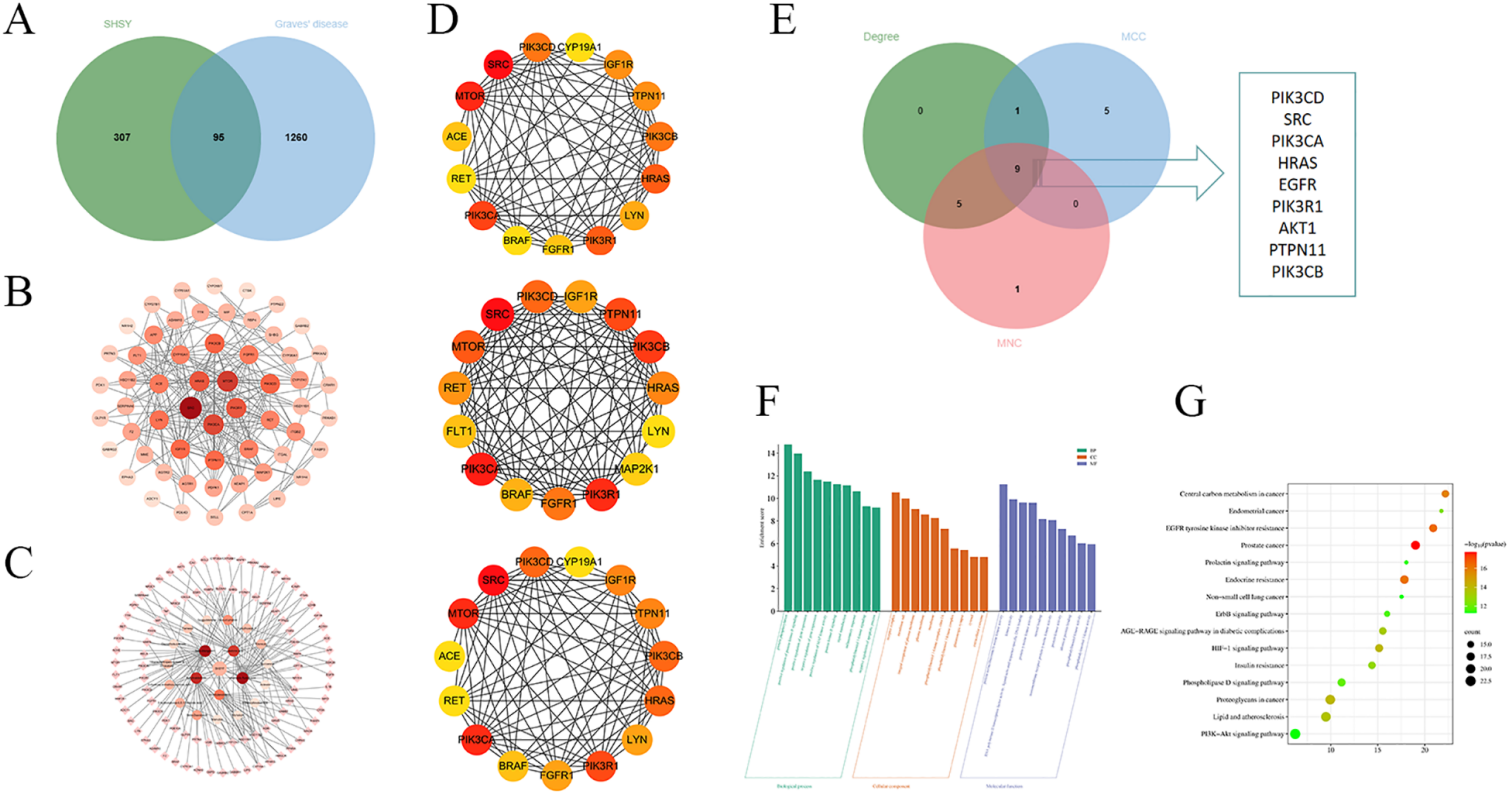
Targets of SHSY in protecting against GD. **(A)** Veen diagram of common targets between GD and SHSY. **(B)** PPI network related to GD and SHSY. Nodes represent proteins (The colors from pink to red represent the degree of binding between proteins). Edge represents protein-protein association. **(C)** SHSY - GD - common targets network.Circles represent blood-entry components of SHSY (The colors from pink to red represent the degree of binding between proteins), and diamonds represent common targets of SHSY and GD. **(D)** PPI network analyzed in Degree, MCC, and MNC modes. **(E)** Overlapping targets in Degree, MCC, and MNC modes. **(F)** GO enrichment analysis of SHSY targets in treating GD. **(G)** KEGG pathway enrichment analysis of SHSY targets in treating GD.

Target prediction was conducted using the Swiss Target Prediction database, revealing 396 targets for the blood-entry components of SHSY. Additionally, 1,355 GD-related targets were identified from the OMIM, Disgenet, Drugbank, and GeneCards databases. These data were used to construct a Venn diagram in Bioinformatics, yielding 95 common targets (**Figure 3A**). These common targets were analyzed in the STRING database for PPI network analysis, and the results were visualized in a PPI network graph using Cytoscape 3.7.2 (**Figure 3B**), which displayed 85 nodes and 383 edges. Further analysis of the blood-entry components and disease common targets in Cytoscape 3.7.2 resulted in a network of 116 nodes and 165 edges, differentiated by size and color based on Degree value (**Figure 3C**). Using the CytoHub plugin to calculate by Degree, MCC, and MNC, the top 15 intersections were examined to identify core targets of SHSY for GD, resulting in nine intersecting targets: PIK3CD, SRC, PIK3CA, HRAS, EGFR, PIK3R1, AKT1, PTPN11, and PIK3CB, respectively (**Figures 3D, E**).

GO enrichment analysis and KEGG pathway analysis were conducted using the DAVID database for the common targets of SHSY and GD. The analysis resulted in 431 entries for GO biological processes (BP), 102 entries for molecular functions (MF), and 62 entries for cellular components (CC). The top ten significant entries from each category were selected for visualization (**Figure 3F**). The results indicate that BP are predominantly enriched in protein phosphorylation, positive regulation of protein kinase B signaling, and positive regulation of gene expression. CC are primarily associated with the receptor complex, integral component of plasma membrane, and phosphatidylinositol 3-kinase complex, including class IA. MF mainly include protein serine/threonine/tyrosine kinase activity, transmembrane receptor protein tyrosine kinase activity, protein tyrosine kinase activity, and RNA polymerase II transcription factor activity, ligand-activated sequence-specific DNA binding. A total of 158 signaling pathways were identified, with the top ten pathways visualized and presented in bubble diagrams (**Figure 3G**), highlighting significant enrichment in EGFR tyrosine kinase inhibitor resistance, HIF-1 signaling pathway, and PI3K/Akt signaling pathway.

### Molecular docking between the blood-entry components of SHSY and the core targets

**Figure 4:**
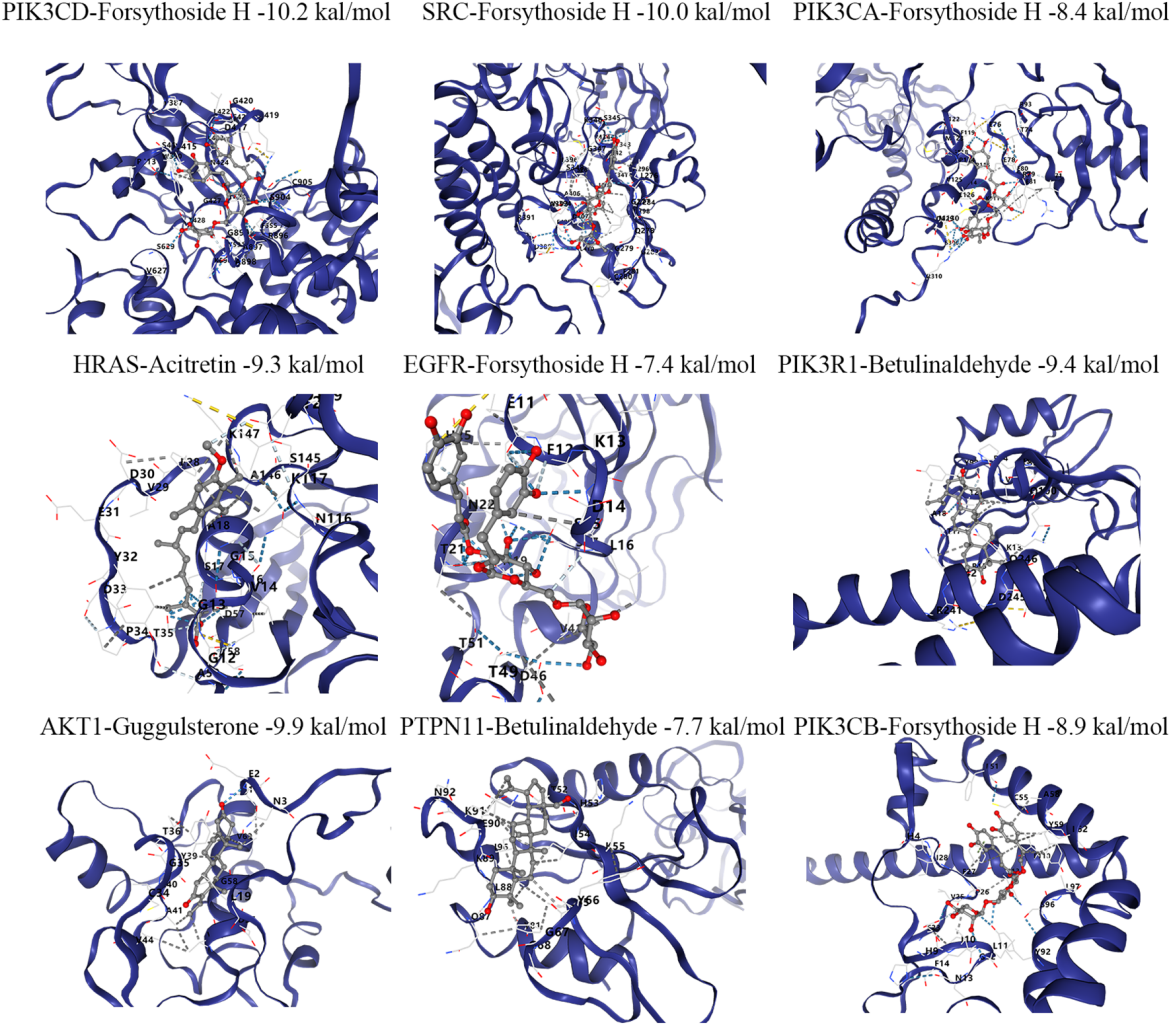
Molecular docking diagram of blood-entry components and key targets in SHSY.

Molecular docking was utilized to predict the binding of SHSY’s active ingredients to its core targets. The strongest bindings for each target were selected for display (**Figure 4**). It was observed that most blood-entry components effectively bound to the core targets of SHSY for GD treatment. Notably, Guggulsterone, Betulinaldehyde, and Forsythoside H exhibited stronger binding to several targets, with Forsythoside H being the most potent.

### Analysis of IHC results

**Figure 5:**
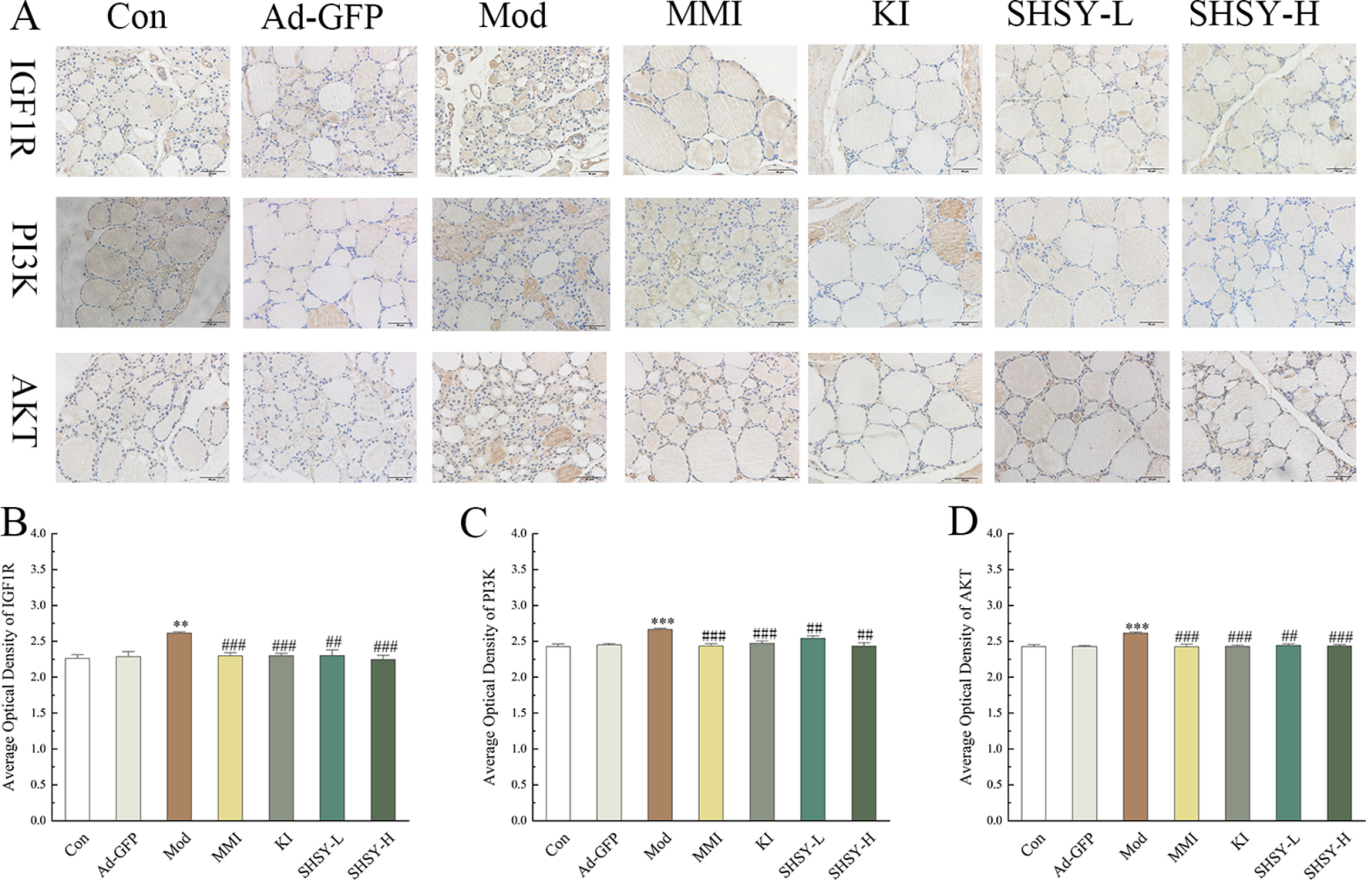
IHC results of IGF1R, PI3K, and AKT in GD mice. **(A)** Representative IHC for IGF1R, PI3K, and AKT. **(B–D)** Bar graph displaying OD values of IGF1R, PI3K, and AKT across different groups. Compared to the Con group, **P<0.01, ***P<0.001; compared to the Mod group, ^##^ *P*<0.01, ^###^ *P*<0.001.

IHC was performed on thyroid tissues for IGF1R, PI3K, and AKT (**Figure 5**). The results indicated that the OD value of IGF1R staining was higher in the Mod group (2.61 ± 0.016) compared to the Con group (2.26 ± 0.063, *P*<0.001), and decreased to 2.30 ± 0.044, 2.30 ± 0.029, 2.30 ± 0.073, and 2.24 ± 0.053 respectively after treatment with MMI, KI, SHSY-L, SHSY-H. Statistical significance was noted as *P*<0.01 between the Mod and SHSY-L groups, with all other comparisons showing *P*<0.001; no significant difference was observed between the SHSY-L and SHSY-H groups. The OD value for PI3K staining also increased in the Mod group (2.67 ± 0.014) compared with the Con group (2.43 ± 0.031, *P*<0.001), and decreased to 2.44 ± 0.028, 2.47 ± 0.029, 2.54 ± 0.032, 2.43 ± 0.048 respectively after treatments. Differences between groups were consistent with the IGF1R results, but the SHSY-H group showed a slightly better effect than the SHSY-L group (*P*<0.05). The OD value for AKT staining increased in the Mod group (2.61 ± 0.016) compared with the Con group (2.43 ± 0.026, *P*<0.001), and decreased to 2.42 ± 0.034, 2.43 ± 0.019, 2.44 ± 0.026, 2.44 ± 0.018 respectively after treatments, with no significant differences noted between the SHSY-L and SHSY-H groups.

## Discussion

In this study, we confirmed that SHSY effectively reduces serum T4 and TRAb levels in mice with GD and alleviates thyroid gland pathology. Besides, in present study, we didn’t find statistically significant difference between the effect of SHSY groups and MMI group and KI group in reducing T4 and TRAb in GD mice, indicating that SHSY has similar therapeutic effect. We also conducted a study on the efficacy of SHSY and MMI in GD patients, and found that SHSY was close to MMI in improving thyroid function in GD patients (data not yet published).It remains unclear whether long-term treatment with SHSY can have a similar effect as ATDs and KIs in relieving GD. It is also interesting to see what the long-term efficacy of different doses of SHSY will be.

Utilizing UPLC-Q-TOF-MS technology, we identified the blood-entry components of SHSY. Network pharmacological analysis was conducted to predict potential targets and related pathways, with these predictions further substantiated through molecular docking and IHC, thus enhancing the credibility of our findings.

A total of 19 blood-entry components of SHSY were detected and analysing network pharmacology. The potential core targets identified for the treatment of GD included PIK3CD, SRC, PIK3CA, HRAS, EGFR, PIK3R1, AKT1, PTPN11, and PIK3CB. Molecular docking results indicated effective binding of most blood-entry components to these core targets. Notably, Guggulsterone, Betulinaldehyde, Forsythoside H, and several other components demonstrated strong binding affinities to multiple targets, suggesting they are key contributors to SHSY’ s therapeutic effects in GD.

Guggulsterone, a plant steroid, is utilized in treating various conditions such as inflammation, hyperlipidaemia, and thyroid disorders (19). It modulates anti-apoptotic and pro-inflammatory genes through signaling pathways including JAK/STAT, NF-kB, and PI3K/Akt, thus influencing growth and inflammatory responses (20). Research indicates that myrcophenostatin protects against COPD-associated inflammation and emphysema by regulating genes for pro-inflammatory mediators like TNF-α, IL-1β, G-CSF, and KC. This regulation not only prolongs survival but also suppresses the expression of inflammatory mediators including IL-1β, IL-6 and TNF-α, and mitigates LPS-induced liver injury (21, 22). Betulinaldehyde, a triterpenoid with diverse biological activities, inhibits proliferation, migration, and phenotypic transformation of vascular endothelial cells and downregulates MMP9 expression, contributing to cardiovascular disease treatment (23). It was observed that betulinaldehyde suppresses the activation of Akt, MAPK, and STAT3 signaling pathways in A549 cells in a time-dependent manner and modulates intracellular autophagy levels, significantly inhibiting tumor activity in A549 cells, suggesting its potential as an effective adjuvant therapy for lung cancer (24). Although research on Forsythoside H is limited, existing studies show it possesses potent antioxidant and antimicrobial properties (25, 26).

Further investigation through KEGG pathway enrichment analysis revealed that SHSY’s treatment of GD primarily involves pathways like EGFR tyrosine kinase inhibitor resistance, HIF-1 signaling, and PI3K/Akt signaling. Notably, the PI3K/Akt signaling pathway includes five core targets for GD treatment by SHSY, highlighting its potential as a key pathway for therapeutic effects.

The PI3K/Akt signaling pathway is a crucial molecular cascade involved in cell growth and is closely linked to the development of various tumors. It is activated by aberrant receptor tyrosine kinases, promoting cell and vascular proliferation (27). Recently, the connection between the PI3K/Akt signaling pathway and thyroid disease has garnered increased attention. The thyrotropin receptor (TSHR), a G protein-coupled receptor, triggers the PI3K/Akt signaling cascade. In thyroid cells and some cancers, TSHR signaling stimulates cell proliferation via cAMP- and PI3K/Akt-dependent pathways (28). The PI3K/Akt signaling pathway is pivotal in the activation of T follicular helper (Tfh) cells, where PI3K and Akt regulate various downstream effectors leading to Tfh cell differentiation (29). This pathway is also crucial for the development of regulatory T (Treg) cells (30). GD is a prevalent clinical autoimmune thyroid disease characterized by the production of TRAb, which promote the proliferation of thyroid cells and the synthesis and release of thyroid hormones, thereby precipitating GD (31). Tfh cells are implicated in TRAb production, while Treg cells and their subset, follicular regulatory T cells, mediate Tfh and B cell interactions and regulate the germinal center, thus mitigating the autoimmune response (32). These observations underscore the significant role of the PI3K/Akt signaling pathway in the pathogenesis of GD.

The PI3K/Akt signaling pathway includes the IGF1R protein, a transmembrane receptor with tyrosine kinase activity that is crucial for the regulation of growth, development, and metabolism. Overexpression of IGF1R in cancer enhances cell survival and proliferation, and inhibits apoptosis (33, 34). Activation of IGF1R leads to phosphorylation of the intracellular adapter protein IRS, which then recruits and phosphorylates PI3K. This, in turn, phosphorylates AKT, regulating the PI3K/Akt signaling pathway and contributing to the development of GD (35, 36). Our IHC findings revealed that IGF1R, PI3K, and AKT protein levels were upregulated in the thyroid tissues of GD mice, and their expression was downregulated after treatment. These results support the hypothesis that SHSY impacts the IGF1R/PI3K/Akt signaling pathway by targeting multiple components, thereby exerting a therapeutic effect on GD.

This study has certain limitations. As an iodine-rich Chinese herbal compound with a complex composition, SHSY has the potential to act through multiple pathways. However, this study specifically focused on the role of the IGF1R/PI3K/Akt signaling pathway without exploring other potential signaling pathways and also lacked validation at the cellular level and clinical practice. In addition, a bias due to using a single herbal formula source from one hospital may also exist. Therefore, further studies are required to fully elucidate its effect and mechanisms in the treatment of GD.

## Conclusions

In this study, we identified a total of 19 blood-entry components of SHSY, which were further analyzed using network pharmacological and molecular docking technology, and verified through IHC. The results demonstrated that SHSY modulates the IGF1R/PI3K/Akt signaling pathway by down-regulating the protein levels of IGF1R, PI3K, and AKT. It promotes apoptosis and regulates the immune responses of thyroid cells, thereby exerting a therapeutic effect on GD.

## Data Availability

The original contributions presented in the study are included in the article. Further inquiries can be directed to the corresponding authors.
